# Analysis of Risk Factors in Global Software Development: A Cross-Continental Study Using Modified Firefly Algorithm

**DOI:** 10.1155/2022/4936748

**Published:** 2022-06-06

**Authors:** Asim Iftikhar, Syed Mubashir Ali, Muhammad Alam, Shahrulniza Musa, Mazliham Mohd Su'ud

**Affiliations:** ^1^College of Computer Science & Info. Sys, Institute of Business Management (IoBM), Korangi Creek, Karachi, Pakistan; ^2^Malaysian Institute of Information Technology, Universiti Kuala Lumpur, (UniKL MIIT), Kuala Lumpur, Malaysia; ^3^College of Computing and Information Sciences, Karachi Institute of Economics and Technology, PAF-KIET, Karachi, Pakistan; ^4^Riphah Institute of System Engineering (RISE), Faculty of Computing, Riphah International University, Islamabad, Pakistan; ^5^Multimedia University, (MMU), Cyberjaya, Malaysia; ^6^Malaysian France Institute, Universiti Kuala Lumpur, (UniKL MFI), Kuala Lumpur, Malaysia

## Abstract

In today's competitive world, software organizations are moving towards global software development (GSD). This became even more significant in times such as COVID-19 pandemic, where team members residing in different geographical locations and from different cultures had to work from home to carry on their tasks and responsibilities as travelling was restricted. These teams are distributed in nature and work on the same set of goals and objectives. Some of the key challenges which software practitioners face in GSD environment are cultural differences, communication issues, use of different software models, temporal and spatial distance, and risk factors. Risks can be considered as a biggest challenge of other challenges, but not many researchers have addressed risks related to time, cost, and resources. In this research paper, a comprehensive analysis of software project risk factors in GSD environment has been performed. Based on the literature review, 54 risk factors were identified in the context of software development. These were further classified by practitioners into three dimensions, i.e., time, cost, and resource. A Pareto analysis has been performed to discover the most important risk factors, which could have bad impact on software projects. Furthermore, a modified firefly algorithm has been designed and implemented to evaluate and prioritize the pertinent risk factors obtained after the Pareto analysis. All important risks have been prioritized according to the fitness values of individual risks. The top three risks are “failure to provide resources,” “cultural differences of participants,” and “inadequately trained development team members.”

## 1. Introduction

In the last 2 decades, the world has changed significantly [1]. The exponential advancement in technology has resulted in exchanging information among peers more efficiently and effectively [2]. In the past, we had to walk our way to meet or to have a conversation with someone, but now we can communicate easily using mobile devices. All of this is not a result of high-rise structures, instead a result of technological advancement. Moreover, the field of software development also witnessed a massive and rapid change around the world to embrace the needs of their clients. In order to have more advantages, some software firms have moved from co-located environment to distributed environment [3]. In the last decade, there has been a rise in trend among the software firms to move towards distributed software development, in order to find low-cost and skilled resources [4]. As a result, software development has become diverse, multisite, and globally distributed, and this is also called global software development (GSD). However, software professionals also face some challenges, such as social and cultural diversity in globally distributed team while performing some tasks [5].

GSD is also known as offshore software development or outsourced software development. From the past two decades, software outsourcing which is a corporate-level strategy has been adopted by numerous firms [6]. The software outsourcing model is used in order to produce high-quality software at a low cost [7, 8]. But it is rather easier said than done when it comes to adopting GSD for software projects due to a number of barriers [9]. Globalization, as a result of technological advancement, results in cultural heterogeneity and diversity [10, 11]. People, businesses, and various organizations are investing a lot of capital in order to understand and overcome barriers that comes with cross-cultural teams. The barriers of GSD, if catered properly, can ensure timely and successful implementation of software projects [12].

**Figure 1:**
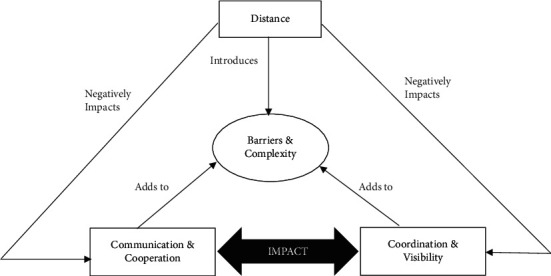
GSD issues [19].

GSD is a contemporary model. In GSD, developers within a team are spread cross-borders. The team members keep exchanging information and work together even after being in diverse time zones and organizational boundaries [13]. Although it becomes very difficult for the team members to work in a GSD setting, it has got acceptance by the industry due to restricted travel freedom as well as increasing travelling cost. Cheap skilled labour, better productivity, work efficiency, economic benefit, etc., are some of the key benefits of GSD [3, 14, 15]. Keeping aside all these benefits, people working in a GSD environment face many difficulties such as lack of communication, strategic issues, project management issues, and cross-cultural backgrounds of the team [16–18]. Various issues related to GSD are depicted in Figure 1.

We can divide the GSD projects into 2 categories, offshore and onshore. The reason for the failure of offshore projects is physical time constraints and cultural differences. Not just time and culture but communication gap is also a major issue faced by the offshore and onshore teams [20, 21]. This can result into less productivity, poor project quality, and decreased efficiency [22, 23]. Therefore, in order to harness the advantages of GSD, it is imperative to look into the risk factors that come with it and mitigate those risks before starting any project that involves distributed teams [19, 22–24].

There are various risks associated with GSD projects. If the team is located in different countries around the world or in different regions globally, they can face obstacles such as geographical risk, language barrier, and even weather conditions [25, 26].

Majority of the software organizations are at risk in the GSD environment. They tend to reduce the risks using standard risk management tools. However, they realize that these tools are not competent enough to cater to the crucial and critical characteristics of GSD. Therefore, this research aims at identifying and prioritizing the most pertinent risk factors for GSD. For this purpose, we have employed modified firefly algorithm (MFA). Firefly algorithm (FA) is a machine learning (ML) technique which is getting popular these days due to their ability to deal with unstructured data [27]. Simple firefly algorithm (FA) does not provide any way to validate its results i.e., fitness scores. Therefore, MFA has been designed and implemented that calculates the variance of all fitness values of risks with respect to time, cost, and resource to make sure that fitness values obtained are reliable.

This paper comprises 7 sections.  Section 1 gives the introduction to this research. Literature review is presented in Section 2. Research methodology is elaborated in Section 3.  Section 4 provides the results and discussion. Research implications are discussed in Section 5.  Section 6 highlights limitation and future research directions. The last section concludes this research study.

## 2. Literature Review

We can define risk as the possibility of having a negative or positive effect on an occurring event [28]. Management strategies have many critical functions, one of which is known as risk management. It looks into the loopholes of the system, by the internal control mechanism, which has tested procedures and practices to manage the loopholes. It also helps to identify, analyze, evaluate, inspect, and handle the risk [29, 30].

### 2.1. Risk Management

**Figure 2:**
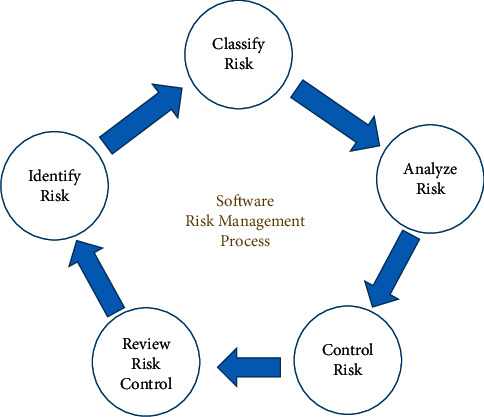
Risk management process.

Project management has many branches; one of the most important is risk management. It plays a very vital role in project management. It prevents the risk that can affect the desired outcomes and results. Preventive measures are taken by small- and medium-sized enterprises (SME) to minimize the risk [31]. To look for any undesired or unexpected errors in a project, a well-planned risk management strategy is always needed by SME [32]. There are five risk management steps in risk management process [29, 30, 33, 34] (see Figure 2):

  Step 1: Identify the risk. The task of the team is to highlight risks that might affect the project, for which various techniques are used, out of which the first is to maintain a project risk register.
  Step 2: Classify the risk. Different risks are grouped together according to their estimated cost or likely impact and probability of occurrence. For example, credit risk, is classified according to the likelihood of the collection of repayments from the debtor.
  Step 3: Analyze the risk. After identification of risk, next important step is to analyze the consequence of each risk, where nature of risk and its capacity to affect project result are determined. This information is also fed into the project risk register.
  Step 4: Control the risk. After risk analysis, risk control takes place. It is the method by which software firms evaluate risks and take action to mitigate or eliminate such risks or threats. This is known as the risk control hierarchy, i.e., eliminating the hazard is the most effective control, which must always be aimed at.
  Step 5: Review risk control. It is to ensure that the control measures that have been implemented are effective and efficient. It must be reviewed and revised to make sure that they work as planned to determine if any remedial action needs to be taken immediately.

### 2.2. Relevant Work

The authors of [35] applied FA to optimize the established parameters of varying estimation models. They used it in comparison to other metaheuristic instructions like genetic algorithm (GA) and particle swarm optimization (PSO) algorithm. Models named previously are the variations constructive cost model also known as COCOMO. Authors claim that their experimental results show that FA is more precise and causes decrease in error over the other the GA and PSO.

In [36], researchers proposed a hybrid software fault-prediction (SFP) model that was constructed using FA and artificial neural network (ANN), along with an observational differentiation of GA and PSO grounded evolutionary techniques. From the PROMISE repository, they took seven different faulty data sets to perform their studies. Based on these data, they claim that the results are showing that the FA-ANN model outperformed GA and PSO ANN fault-prediction models. The authors concluded that (FA-ANN)-based model does not cause any as such hindrance as shown by other models and proved to be statistically significant. On the other side, this proposed model reduces the software cost and enhances the final product quality.

In [37], the authors made some alterations in the FA to the portfolio optimization problem that gave them a satisfactory exploitation/exploration balance in the portfolio. They call this an upgraded FA. The authors claim that the enhanced or upgraded algorithm showed to be consistently better than the original, for all portfolio problems. They made this conclusion after comparing their upgraded FA algorithm with five previous results of optimization metaheuristics from the publications. They are confident that the upgraded firefly algorithm is by far better than previous measurements of required performance indicators.

The authors of [38] argue that among many of the effort-prediction models available, making the choice can be a hard for the project managers. Their paper researches the possibility to improve the accuracy of software cost estimations. They accomplish this by using a FA with the ANN models used for cost predictions. We are talking about cost estimation as compared to the PSO. They used functional link ANN models with radial basis function network. They argue based on their results that ANN models are better for data processing when incorporated with the FA in addition to the intuitionistic fuzzy C-means.

### 2.3. Project Time, Cost, and Resource Risk Dimensions

**Figure 3:**
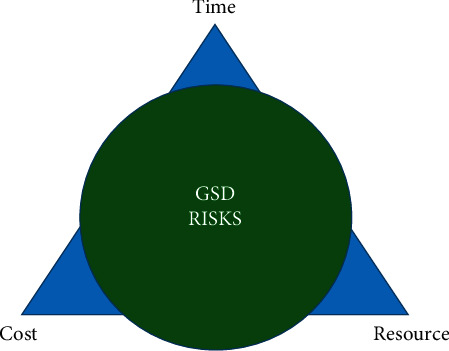
Three risk dimensions of GSD projects.

The risks in software development projects can be categorized into time, cost, and resource or a combination of these by examining risk sources [39]. Project time, cost, and resource are the main concerns of project management that may negatively influence one of more aspects of project performance in the GSD environment (see Figure 3).

#### 2.3.1. Time-Related Risk Factors

It will not be an exaggeration to say that “the time is what defines the success of any project.” The project managers face 2 kinds of challenges when it comes to time-related risk factors: (1) number of adjustments needed during the project's execution; (2) time spent on unessential activities [40]. How to deal with these two challenges in a simple way is by having a well-defined project plan and timeline beforehand and then following it. Having a well-defined project plan is needed for an effective time management, and project managers should make such project plan and timeline to reduce the above-mentioned two risk factors.

#### 2.3.2. Cost-Related Risk Factors

The cost is another constraint in addition to time which can be easily measured. Just like the timeline of the project, the cost structure for the project also has to be estimated beforehand as accurately as possible [41]. Cost is simply the amount of money that should be invested in a particular project to finish it. It is also known as budget for the project. Knowing the cost structure beforehand gives one a baseline against which one can measure and monitor project's actual cost while the project is in progress. This allows the project manager to avoid facing any surprise costs which can pop up during the project.

#### 2.3.3. Resource-Related Risk Factors

The resources in a project are of two kinds: one is the human and the other is material. The project managers should take the availability of both into consideration. This constraint is greatly dependent upon the cost structure: The more money one has, the more material resources one can buy and higher quality expertise can be hired as well. Of course, money cannot solve the problem of availability and accessibility in the market; hence, a project manager should keep such challenges in mind when figuring out the timeline and the cost structure.

### 2.4. Software Development Risks

A sophisticated and structured literature review was conducted in view of the risks faced during software development, management, and assessment of risks. Survey of the literature review has resulted in the identification of fifty-four probable risk factors related to software development industry. Identified risk factors along with the references are shown in Table 1.

## 3. Research Methodology

**Figure 4:**
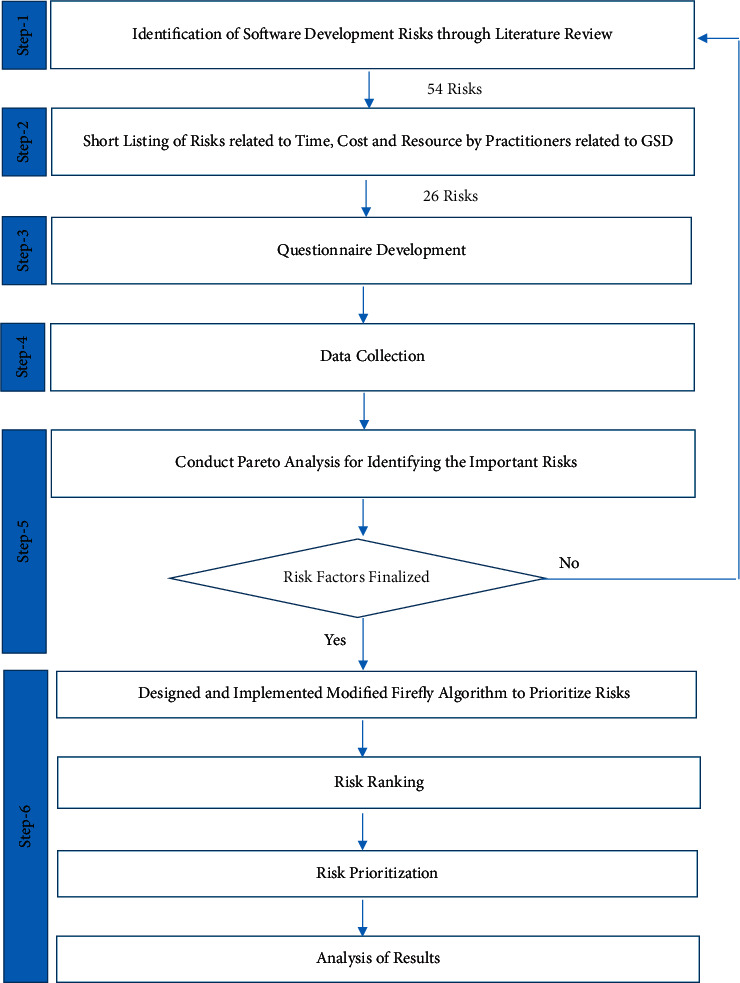
Research framework.

For the attainment of the objectives of the research and the analysis of the pertinent risk factors which are related to the GSD environment, a systematic research methodology is followed (see Figure 4).

### 3.1. Research Design

**Figure 5:**
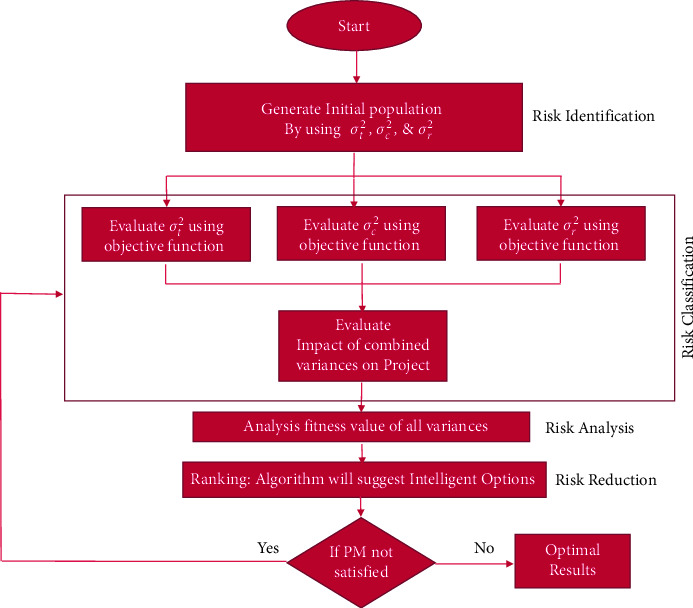
MFA block diagram.

This research will employ experimental as well as simulation-based research design. First, an exhaustive literature review has been conducted to identify the GSD risks related to project cost, time, and resources. Later, three hundred forty-two large- and medium-sized software houses from the US, Pakistan, and Australia had been shortlisted through convenience sampling technique to collect the data:

  Step 1: identification of software development risks
  In the first step, fifty-four risk factors relevant to the software project in a software development (SD) environment were identified after extensive literature review. Search keywords including but not limited to “risk management in SD,” “software risk management using ML,” “project management risks,” and “risk assessment in distributed projects” were used to search databases such as the Google Scholar, Science Direct, and Web of Science.
  Step 2: shortlisting of risks by practitioners
  In the next step, the list of fifty-four risk factors was given to industry experts working in GSD environment to further remove duplicate risk factors and finalize the risk factors relevant to GSD. This resulted in a reduced list of twenty-six risks factors related to cost, time, and resources that can affect GSD projects negatively (see Table 2). 6 industry experts gave their feedback at this stage, and their short profiles are presented (see Table 3).
  Step 3: questionnaire development
  Once the practitioners have shortlisted risk factors, a survey questionnaire was developed which was mapped to all twenty-six risk factors relevant to GSD.
  Step 4: data collection
  In this step, data were collected by sending the questionnaire to seven hundred sixty large- and medium-sized software houses based in the US, Pakistan, and Australia. Project managers, team leaders, system analysts, and business analysts are the respondents of this research, whose active participation concluded this research.
  Step 5: the most important risk identification using Pareto analysis
  In this step, a Pareto analysis has been performed to summarize experts' opinions and recognize the important risk factors with respect to time, cost, and resource in the GSD environment. Pareto chart is an industry benchmark. Used not just to pin point the major areas of concern, it also aids management and other decision makers in achieving the solution [48]. Both the bar graph and a line graph are the components that make a Pareto chart. Attributes that are under consideration are represented by the bar. A bar represents risk factors that were identified through the literature survey. The line represents the cumulative percentage of the attributes. In our scenario, the line represents the frequency of expert opinion. The bar in a Pareto chart is always displayed in the descending order, which results in the ease to spot the most common attributes. It highlights the most important risk factors of the software industry in our scenario.
  Step 6: implementation of MFA to prioritize risk
  FA does not provide any way to validate fitness values; therefore, MFA was used to calculate variance of all fitness values of risk with respect to time, cost, and resource to make sure that fitness values obtained are reliable.  Figure 5 shows the framework of MFA.

We initialize the fireflies' population by considering (1).Risk identification will be based on initial population (data) that will be generated through questionnaires.

(1) $$\begin{array}{c} x_{t+1}=x_{t}+\beta_{0}e^{-yr^{2}}+\alpha \varepsilon , \end{array}$$

where *x* is the firefly position in the iteration, *β*_0_*e*^−*yr*^2^^ is attraction between fireflies, and *αε* defines randomization and vector of random numbers.

Fitness values of risks related to project time, cost, and resource will be evaluated from objective function. Risk classification will be done by calculating variance of time, cost, and resource risks and combined variance will also be calculated.

(2) $$\begin{array}{c} FR=\;\frac{1-\left(\left(\sum \text{LVL}\right)/\left(n-\text{TN}\right)\right)+\left(\sum \text{ULVUL}\right)}{1+\;\left(\sum \text{ULVUL}\right)}. \end{array}$$

Here, FR is the fitness value of risk, LVL defines likely and very likely, ULVUL defines unlikely and very unlikely, TN defines total neutral, and *n* defines total no. of responses.

Risk analysis will be based on fitness values of risks which will be calculated using

(3) $$\begin{array}{c} x_{i}=x_{i}+\beta_{0}e^{-yr_{i,j}}\left(xj-xi\right)+\alpha \varepsilon , \end{array}$$

where *x* is the firefly position in the iteration, *β*_0_*e*^−*yr*^2^^(*xj* − *xi*) is attraction between firefly *j* and *i*, and *αε* defines randomization and vector of random numbers.

Risk reduction will be performed by ranking individual risks to prioritize the most important risks.

### 3.2. Data Collection Procedure

Various risks relevant to GSD were assessed by developing a survey questionnaire. The questionnaire had a total of thirty-three questions, out of which 18 questions were addressing 3 categories of GSD risks, namely, time, cost, and resource. The remaining 15 questions were general and open-ended. The survey questions were closed-ended and scored with a 5-point Likert scale from very unlikely to very likely. The questionnaire was circulated to more than seven hundred fifty medium and large software companies based in Australia, Pakistan, and the USA. A total of four hundred sixty responses were received. One hundred eighteen responses were rejected due to missing information. So, a total of three hundred forty-two valid responses were left for analysis. For sample data set, see Tables 4 and 5. Later, a Pareto analysis was carried out to find out the most pertinent risk factors.

## 4. Results and Discussion

This section will first provide numerical illustration of our proposed methodology and secondly will discuss the results.

### 4.1. Numerical Illustration

Our model was applied to software houses from the US, Australia, and Pakistan. In order to achieve the objectives of this study and to analyze the pertinent risk factors associated within GSD, we will use the integrated Pareto and MFA. The reason of using integrated Pareto-MFA is Pareto analysis will help in data reduction, i.e., reducing by short listing the most pertinent risk factors while MFA will enable us to rank them. The proposed method's application is divided into two phases as follows.

**Phase 1 .**

Identification of critical risks using Pareto analysis

**Figure 6:**
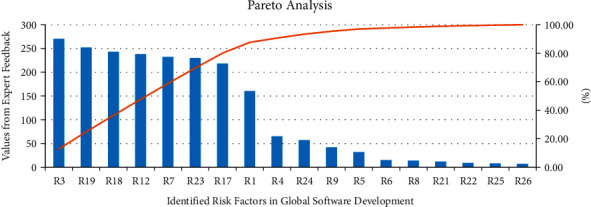
Pareto chart.

In this phase, the data collected from the survey as mentioned in section “Data Collection Procedure” for the shortlisted twenty-six risks were used for conducting the Pareto analysis. Pareto analysis revealed that among the twenty-six shortlisted risks, 7 risk factors are responsible for 80% of the project risk within the GSD. These risk factors are “failure to provide resources,” “cultural differences of participants,” “inadequately trained development team members,” “inappropriate task timings,” “cost overruns,” “inadequate technical resources,” and “lack of balance on the project team.” For Pareto analysis result, see Figure 6.

**Phase 2 .**

Application of MFA

In this phase, MFA was used to evaluate and prioritize the pertinent risk factors obtained after the Pareto analysis. The process is further elaborated as follows:

  Step 1: in this step, the most important risk factors were identified from Pareto analysis with respect to 3 risk dimensions of project, i.e., cost, resources, and time. 7 risk factors were obtained as a result of Pareto analysis (see Table 6).
  Step 2: in this step, MFA was applied on the survey data of the most pertinent risk factors using the objective function given as equation (2) in section “Implementation of Modified Firefly Algorithm to Prioritize Risk”. The fitness scores of each of the risk factors will be used to further calculate the variance in order to evaluate that the resulting scores are consistent. In this research study, the variance was less than 0.01, and therefore, it depicts that our fitness values are consistent and have no outliers. For final fitness scores, variance, and ranking of pertinent risk factors, see Table 7.

### 4.2. Discussion

In this research, we performed comprehensive literature review to identify possible risk factors under 3 dimensions of GSD risks which are time, cost, and resource. Then, professionals were requested to verify the relevance of risk factors and map risk factors to each dimension as well as merge duplicate risks. Moreover, we conducted Pareto analysis to identify the most pertinent risk factors for the GSD projects. And finally, we employed MFA to rank the most pertinent risk factors.

Our study has revealed that “failure to provide resources (R3)” is the most critical risk factor for GSD projects on first rank. This risk indicates that one of the biggest risks in any GSD project is the nonavailability of the required resources. The next ranked risk factor in our analysis is “cultural differences of participants can cause problems like rework, loss of data, confusions, etc., (R19).” Its second position signifies that this GSD risk factor needs serious attention too and has to be treated and taken care of in order to successfully implement GSD projects. When team members are from diverse culture and backgrounds, it becomes a great challenge to have understanding and harmony among all team members [18]. Having good collaboration among project team members is imperative for the smooth implementation of any project in general, and this result shows that it is also a significant risk factor even for GSD projects. The next most important risk factor for GSD project as per our study is “inadequately trained development team members (R18)” which is the third most important risk. As technology is advancing at a rapid pace, continuous training of development team is imperative and it is even more important in case of GSD projects. So, in order for any successful completion of GSD projects, all team members need to be trained periodically so as to mitigate this important risk factor. The fourth most important GSD risk factor as revealed by the study is “inappropriate task timings (R12)” which is about assigning unrealistic deadlines for each task. This risk also needs attention in order to complete the project within the agreed timeline and successfully. “Cost overruns (R7)” comes next in at the fifth position in our analysis of the most important risk factors for GSD. This is such an important risk factor as it can actually derail the whole project as well as the business viability for the software house(s) in the GSD projects. In order to get true financial benefit as well as successful completion of any GSD project, catering to this risk is highly desirable. The sixth and the seventh most important risk factors are “lack of balance on the project team (R17)” and “inadequate technical resources (R23)” which also needs attention of GSD team leads and decision makers in order to successfully implement the GSD projects.

## 5. Research Implications

Various theoretical and practical implications can be observed as a result of this research study. From theoretical perspective, this research has done a significant contribution by identifying and analyzing the most pertinent risk factors associated with GSD with respect to time, cost, and resources. From the methodological standpoint, this research is the first to integrate Pareto analysis and MFA for the purpose of risk assessment in general and GSD in particular. This study enabled us to harness the advantages of both these methods as follows:

- Using Pareto analysis, we were able to identify the risks that creates the most impact on GSD projects
- MFA helped us in evaluating the risk factors and get the most reliable and consistent results

Talking about the research findings, to the best of our knowledge, this is first study which focused on the risk assessment of GSD in cross-continental environment using Pareto and MFA and the seven most pertinent risk factors have been identified and ranked accordingly, which may be taken care of one by one in a GSD environment. From managerial point of view, this study is a significant contribution. The findings of this research study may assist practitioners to realize the risk factors involved in GSD in advance and can guide the top management and policy makers to set the proactive, active, and reactive risk mitigation mechanism to overcome these risks and complete their GSD projects successfully.

## 6. Limitations and Future Directions

The data have been gathered from Pakistan, Australia, and the USA in this research. To expand or widen the scope, other countries will also be included in future. This will help us to understand what are common trends and what are different trends related to GSD in other countries of the world. The results of this study cannot be generalized as the data are collected using convenience sampling as a sampling procedure. Random sampling will perform better than convenience sampling, and this problem will be resolved because results and finding of the study could be generalized.

GSD environment has risks associated with it, and to distinguish those risks, we use ML techniques. In future, other nature-inspired algorithms such as genetic algorithm, particle swarm optimization, and lion optimization algorithms can be used to rank the risks. Moreover, multicriteria decision-making techniques can also be used for ranking the identified risk factors of GSD.

## 7. Conclusion

It is not simple to create or maintain a GSD environment in the field of software engineering. In GSD, distributed software teams are facing many challenges which should be recognized earlier in the development process. Good risk management practices must be incorporated in distributed teams, because you are dealing with practitioners who are from different cultures, time zones, geographical locations, backgrounds, and past project experiences. ML algorithms or techniques give more practical approach than conventional techniques to address risk management. In this study, a comprehensive analysis of software project risk factors in GSD environment has been accomplished. Fifty-four software development project risks factors have been identified from the literature, and these are further shortlisted to twenty-six risks by software practitioners and classified into 3 dimensions: time, cost, and resource. A Pareto analysis that was performed revealed that 7 risk out of twenty-six shortlisted risks are the most important risk factors that could have bad impact on software projects, with respect to project time, cost, and resource in GSD environment. Furthermore, the MFA has been designed and implemented to evaluate and prioritize the pertinent risk factors obtained after the Pareto analysis. All the important risks have been prioritized according to the fitness value of the individual risks.

## Figures and Tables

**Table 1:** List of fifty-four software development risks identified through literature review.

S. no.	Risk factor	Supported references
1	Change in project requirement	[22, 42, 43]
2	Lack of ineffective PM methodology	[42, 44]
3	Inappropriate task timings	[43]
4	Failure to provide resources	[45, 46]
5	Failure in activity estimation and scheduling	[21]
6	Inappropriate planning	[22, 29, 42]
7	Low productivity	[44]
8	Delays in supply	[43, 46]
9	Lack of quality	[22, 29, 46]
10	Failure in requirement capture	[45]
11	Inappropriate design of project	[42–44]
12	Insufficient incentive and motivational system	[45, 46]
13	Lack of cooperation and coordination among team members	[22, 42, 45]
14	Payment issue	[43]
15	Lack of commitment	[43, 45]
16	Mistrust	[22, 45]
17	Project milestones not clearly defined	[42, 44]
18	Inadequate support from top management	[43, 44]
19	Frequent turnover within the project team	[22, 44]
20	Lack of specified skills	[44]
21	Inexperienced project manager	[42–47]
22	Insufficient communication	[22, 29, 42, 44, 45]
23	Incorrect system requirement	[42, 48]
24	Unclear system requirement	[42, 48]
25	System requirement not adequately identified	[42, 48]
26	Lack of motivating attitude	[44]
27	Immature technology	[47]
28	Organization restructuring during the project	[47]
29	Unstable organization environment	[47]
30	Change in organization during the project	[42, 44]
31	Shortfall in supplied components	[43, 46]
32	Adding unnecessary features	[49]
33	Deadline pressure	[49]
34	Wrong documents	[29, 43, 44]
35	Requirement document not shared with distributed team	[21]
36	Lack of common understanding of requirement	[21]
37	Cultural differences of participants	[22, 29, 45]
38	Lack of collaborative office environment	[21]
39	Increased no. of sites	[47]
40	Political state	[42, 45]
41	Social state	[43]
42	Financial condition of target market	[43]
43	Developers lack of motivation	[44]
44	Lack of previous experience	[44]
45	Inadequate estimation of required resources	[42, 46]
46	People maturity	[42]
47	Lack of information security	[43]
48	Project progress not monitored closely enough	[42, 44]
49	Inadequately trained development team members	[22, 42, 44]
50	Failure in process	[44]
51	Use of new technology	[47]
52	Project time estimation error	[46]
53	Insufficient knowledge and expertise	[42, 43, 45]
54	Inappropriate leadership and control	[43]

**Table 2:** List of twenty-six risks related to time, cost, and resource shortlisted by practitioners.

Risk factor	Risk no.	Question	Risk dimension
Lack of ineffective PM methodology	R1	Q13	Time
Inappropriate task timings	R2	Q14	
Failure to provide resources	R3	Q15	
Failure in activity estimation and scheduling	R4	Q17	
Inappropriate planning	R5	Q19	
Unrealistic time estimate	R6	Q20	
Cost overruns	R7	Q26	
Inexperienced project manager	R8	Q27	
Project progress not monitored closely enough	R9	Q28	

Lack of balance on the project team	R10	Q8	Cost
Lack of ineffective PM methodology	R11	Q13	
Inappropriate task timings	R12	Q14	
Failure to provide resources	R13	Q15	
Cost overruns	R14	Q26	
Inexperienced project manager	R15	Q27	
Project progress not monitored closely enough	R16	Q28	

Lack of balance on the project team	R17	Q8	Resource
Inadequately trained development team members	R18	Q10	
Cultural differences of participants	R19	Q11	
Failure to provide resources	R20	Q15	
Lack of cooperation and coordination among team members	R21	Q16	
Loss of key resource(s) that impact the project	R22	Q21	
Inadequate technical resources	R23	Q22	
Lack of appropriately skilled resources	R24	Q23	
Scope creep	R25	Q24	
Project milestones not clearly defined	R26	Q25	

**Table 3:** Industry experts' profile for risk finalization.

Practitioner ID	Industry name	Role	Years of experience
P1	Software products/financial services	Business system analyst	20
P2	Software house	Technical lead	11
P3	Software house	Technical team lead	10
P4	Software products/services	CEO	22
P5	Software house	Chief technology officer	20
P6	Software house	Project manager	18

**Table 4:** Sample data set Part-I (from total of 342 data sets).

Country	Q1	Q2	Q3	Q4	Q5	Q6	Q7	Q8	Q9	Q10	Q11	Q12	Q13	Q14	Q15
AUS	1	1	0	1	1	4	2	3	3	3	3	3	4	4	1
AUS	0	0	0	1	1	2	2	3	3	1	1	3	3	1	1
AUS	2	2	1	1	1	1	2	1	3	3	3	1	3	1	1
AUS	1	1	0	1	1	2	2	1	3	3	3	3	3	1	3
AUS	2	1	0	1	1	2	2	3	3	1	1	1	3	1	1
AUS	3	2	0	1	1	3	2	3	3	1	1	3	3	3	1
PAK	2	1	1	1	1	2	2	3	0	3	3	3	3	2	3
PAK	2	2	2	1	1	1	2	2	2	2	1	0	3	3	3
PAK	3	2	1	1	1	1	2	3	1	1	1	0	1	3	1
PAK	0	0	0	1	1	2	2	3	3	1	1	3	3	1	1
PAK	3	2	0	1	1	2	2	2	1	1	1	3	3	3	1
USA	2	1	0	1	1	1	2	4	3	4	4	3	3	1	0
USA	3	1	0	1	1	1	2	3	2	2	2	3	3	3	3
USA	2	1	1	1	1	2	2	1	4	1	1	3	3	3	0
USA	1	1	0	1	1	2	2	1	3	3	3	3	3	1	3
USA	3	2	1	1	1	3	2	3	3	1	1	3	3	3	1
USA	2	1	0	1	1	2	2	3	3	1	1	1	3	1	1

**Table 5:** Sample Data Set Part-II (from total of 342 Data Set).

Q16	Q17	Q18	Q19	Q20	Q21	Q22	Q23	Q24	Q25	Q26	Q27	Q28	Q29	Q30	Output
3	1	3	4	4	3	3	3	4	4	1	4	4	3	0	3
3	4	3	4	4	3	1	3	4	4	1	4	4	3	0	2
3	3	1	3	3	3	1	3	3	3	1	4	4	1	1	2
3	3	3	4	4	3	1	3	4	4	1	4	3	1	0	0
3	3	3	3	3	3	3	3	4	4	3	3	3	3	0	3
3	1	3	4	4	3	3	3	4	4	1	4	4	3	0	3
4	4	4	3	4	3	3	3	3	4	4	3	3	3	1	3
3	3	3	3	3	3	3	3	3	3	2	3	3	2	0	1
3	3	3	3	3	3	1	2	3	3	3	3	1	2	0	0
4	3	3	4	4	2	3	3	3	3	1	3	3	2	0	1
3	3	1	4	4	3	3	3	3	3	2	3	3	1	1	2
4	3	1	4	4	4	4	3	3	3	2	3	3	2	0	3
2	3	4	4	3	3	3	3	3	3	3	3	2	2	0	2
3	3	3	4	4	3	3	3	3	3	1	3	3	1	1	3
3	3	1	3	3	3	1	3	3	3	1	4	4	1	1	2
3	3	3	3	3	3	3	3	4	4	3	3	3	3	0	3
3	3	3	4	4	3	1	3	4	4	1	4	3	1	0	1

**Table 6:** The most important seven risk factors with respect to GSD.

Risk name	Risk no.
Failure to provide resources	R3
Cultural differences of participants	R19
Inadequately trained development team members	R18
Inappropriate task timings	R12
Cost overruns	R7
Inadequate technical resources	R23
Lack of balance on the project team	R17

**Table 7:** MFA results and final risk ranking.

Risk name	Fitness score	Final rank
Failure to provide resources	0.999021549	1
Cultural differences of participants	0.998734905	2
Inadequately trained development team members	0.998606419	3
Inappropriate task timings	0.996822962	4
Cost overruns	0.995975017	5
Lack of balance on the project team	0.994580603	6
Inadequate technical resources	0.993205793	7

Sum of fitness values	17.56406956	
Mean	0.975781642	
Sum of all squared differences	0.025869716	
Variance	0.001437206	

## Data Availability

The survey data set used to support the results and findings of this study is not been made accessible because the privacy of the data set must be maintained due to PhD studies. However, a sample data set is fused in the paper.

## Supplementary Materials

Supplementary Materials The questionnaire developed in this study covers the items related to the project time, cost, and resource risks that contribute to the overall risks of the global software development projects. On the basis of this questionnaire, data collection was performed. After successful data collection activity, data were analyzed and the desired results were achieved, which are present in the article.

## References

[1] Shah Y. H., Raza M., Ulhaq S. (2012). Communication issues in GSD. *International Journal of Advanced Science and Technology*.

[2] Barros-Justo J. L., Benitti F. B., Molleri J. S. (2021). Risks and risk mitigation in global software development: an update. *Journal of Software: Evolution and Process*.

[3] Al-Zaidi A., Qureshi R. (2017). Global software development geographical distance communication challenges. *The International Arab Journal of Information Technology*.

[4] Jahagirdar R., Bankar S. (2021). PERFORMANCE IN VIRTUAL TEAMS–A CONCEPTUAL OVERVIEW. *Bilingual Research Journal*.

[5] Morrison-Smith S., Ruiz J. (2020). Challenges and barriers in virtual teams: a literature review. *SN Applied Sciences*.

[6] Ali S., Li H., Khan S. U., Abrar M. F., Zhao Y. (2020). Practitioner’s view of barriers to software outsourcing partnership formation: an empirical exploration. *Journal of Software: Evolution and Process*.

[7] Khan S. U., Ali S. (2015). Empirical investigation of success factors for establishing software outsourcing partnership from vendor’s perspective. *Proceedings of the Pakistan Academy of Sciences*.

[8] Ali S., Khan S. U. Critical success Factors for Software Outsourcing Partnership (SOP): A Systematic Literature Review.

[9] Iftikhar A., Musa S., Alam M., Su’ud M. M., Ali S. M. A Survey of Soft Computing Applications in Global Software Development.

[10] Marinho M., Luna A., Beecham S. (2018). Global software development: practices for cultural differences. *International Conference on Product-Focused Software Process Improvement*.

[11] Bhatti M. W., Ahsan A. (2021). Effective communication among globally distributed software development teams. *Research Anthology on Recent Trends, Tools, and Implications of Computer Programming*.

[12] Richardson I., Casey V., McCaffery F., Burton J., Beecham S. (2012). A process framework for global software engineering teams. *Information and Software Technology*.

[13] Hassan M., Hussain M., Irfan M. A policy recommendations framework to resolve global software development issues.

[14] Azeem M. A., Kamal T., Hamza M. (2019). Towards the successful requirements change management in the domain of offshore software development outsourcing: preliminary results. *International Journal of Computing and Digital Systems*.

[15] Leitão Junior N. A Theory of Communication in Distributed Software Development Teams.

[16] Yaseen M., Ali Z. (2019). Success factors during requirements implementation in global software development: a systematic literature review. *International Journal of Computer Systems Science and Engineering*.

[17] Yaseen M., Ali M., Ur A., Nabi S., Khan S., Bacha M. (2020). Practices for effective software project management in global software development: a systematic literature review. *International Journal of Computer Application*.

[18] Iftikhar A., Alam M., Musa S., Su’ud M. M. Trust Development in virtual teams to implement global software development (GSD): a structured approach to overcome communication barriers.

[19] Casey V. (2010). Imparting the importance of culture to global software development. *ACM inroads*.

[20] Karlsen J. T., Sæther H. S., Oorschot K. E. V., Vaagaasar A. L. (2021). Managing trust and control when offshoring information systems development projects by adjusting project goals. *International Journal of Technology Management*.

[21] Verner J. M., Brereton O. P., Kitchenham B. A., Turner M., Niazi M. (2014). Risks and risk mitigation in global software development: a tertiary study. *Information and Software Technology*.

[22] Fabriek M., van den Brand M., Brinkkemper S., Harmsen F., Helms R. Reasons for Success and Failure in Offshore Software Development Projects.

[23] Casey V. Leveraging or Exploiting Cultural Difference?.

[24] Hossain E., Babar M. A., Paik H., Verner J. Risk identification and mitigation processes for using scrum in global software development: a conceptual framework.

[25] Galli B. J. (2018). Addressing risks in global software development and outsourcing. *International Journal of Risk and Contingency Management*.

[26] Khan J. A., Khan S. U. R., Iqbal J., Rehman I. U. (2021). Empirical investigation about the factors affecting the cost estimation in global software development context. *IEEE Access*.

[27] Iftikhar A., Alam M., Ahmed R., Musa S., Su’ud M. M. (2021). Risk prediction by using artificial neural network in global software development. *Computational Intelligence and Neuroscience*.

[28] Tavares B. G., da Silva C. E. S., de Souza A. D. (2019). Risk management analysis in Scrum software projects. *International Transactions in Operational Research*.

[29] Chadli S. Y., Idri A., Fernandez-Aleman J. L., Ros J. N., Toval A. Identifying Risks of Software Project Management in Global Software Development: An Integrative Framework.

[30] Iftikhar A., Musa S., Alam M., Mohd Su’ud M., Mubashir Ali S. (2018). Application of soft computing techniques in global software development: state-of-the-art review. *International Journal of Engineering & Technology*.

[31] Vichova K., Taraba P., Belantova T. (2020). Risk management of the project and the use of software in SME. *WSEAS Transactions on Business and Economics*.

[32] Verbano C., Venturini K. (2013). Managing risks in SMEs: a literature review and research agenda. *Journal of Technology Management and Innovation*.

[33] Sousa A. O. (2021). Assessing risks in software projects through machine learning approaches.

[34] Iftikhar A., Musa S., Alam M., Su’ud M. M. (2020). Artificial intelligence based risk management in global software development: a proposed architecture to reduce risk by using time, budget and resources constraints. *Journal of Computational and Theoretical Nanoscience*.

[35] Ghatasheh N., Faris H., Aljarah I., Al-Sayyed R. M. (2019). Optimizing Software Effort Estimation Models Using Firefly Algorithm. https://arxiv.org/abs/1903.02079.

[36] Arora I., Saha A. (2018). Software fault prediction using firefly algorithm. *International Journal of Intelligent Engineering Informatics*.

[37] Tuba M., Bacanin N. Upgraded firefly algorithm for portfolio optimization problem.

[38] Kaushik A., Tayal D. K., Yadav K., Kaur A. (2016). Integrating firefly algorithm in artificial neural network models for accurate software cost predictions. *Journal of Software: Evolution and Process*.

[39] Bilal M., Gani A., Liaqat M., Bashir N., Malik N. (2020). Risk assessment across life cycle phases for small and medium software projects. *Journal of Engineering Science & Technology*.

[40] Muriana C., Vizzini G. (2017). Project risk management: a deterministic quantitative technique for assessment and mitigation. *International Journal of Project Management*.

[41] Karimi T., Fathi M., Yahyazade Y. (2020). Developing a risk management model for banking software development projects based on fuzzy inference system. *Journal of Optimization in Industrial Engineering*.

[42] Yong H., Juhua C., Zhenbang R., Liu M., Kang X. A neural networks approach for software risk analysis.

[43] Ghaffari M., Sheikhahmadi F., Safakish G. (2014). Modeling and risk analysis of virtual project team through project life cycle with fuzzy approach. *Computers & Industrial Engineering*.

[44] Nieto-Morote A., Ruz-Vila F. (2011). A fuzzy approach to construction project risk assessment. *International Journal of Project Management*.

[45] Reed A. H., Knight L. V. (2010). Project risk differences between virtual and co-located teams. *Journal of Computer Information Systems*.

[46] Christiansen T., Wuttidittachotti P., Prakancharoen S., Vallipakorn S. A. (2015). Prediction of risk factors of software development project by using multiple logistic regression. *ARPN Journal of Engineering and Applied Sciences*.

[47] Hu Y., Zhang X., Ngai E. W. T., Cai R., Liu M. (2013). Software project risk analysis using Bayesian networks with causality constraints. *Decision Support Systems*.

[48] Khan S. A., Kaviani M. A., Galli B. J., Ishtiaq P. (2019). Application of Continuous Improvement Techniques to Improve Organization Performance: A Case Study. *International Journal of Lean Six Sigma*.

[49] Bhatia N., Kapoor N. (2011). Fuzzy cognitive map based approach for software quality risk analysis. *ACM SIGSOFT - Software Engineering Notes*.

