# Supplementary material for: Analysis of Risk Factors in Global Software Development: A Cross-Continental Study Using Modified Firefly Algorithm
Source: Comput Intell Neurosci. 2022 Jun 6;2022:4936748. doi: 10.1155/2022/4936748 (PMC9192222; doi:10.1155/2022/4936748)
Supplement: Supplementary Materials — The questionnaire developed in this study covers the items related to the project time, cost, and resource risks that contribute to the overall risks of the global software development projects. On the basis of this questionnaire, data collection was performed. After successful data collection activity, data were analyzed and the desired results were achieved, which are present in the article. [file 4936748.f1.docx]

**Survey Questionnaire**

Name: __________________________________

Designation: __________________________________

Company Name: ___________________________________

No. of years since founded: __________________________

1. In how many geographical locations or time zones is your project team generally located?
   1. 1-2 B 3-4 C. 4-5 D. 6 or more
2. What percentage of your team is generally located offshore in different time zone(s)?
   1. < 20% B 20-40% C. 40-60% D. > 60%
3. What is the time difference between onsite and the farthest offshore team?
   1. 1 Hr B 3Hrs C. 3-6 Hrs D. More than 6 Hrs
4. Do you use any central document repository to share documents between geographically split team?
   1. Yes B. No
5. Do you use any project management tool for distributed teams to assign tasks and track progress?
   1. Yes B. No
6. What is the average experience of team members in desired area?
   1. Less than 1 Yr B 1-5 Yrs C. 5-10 Yrs D. More than 10 Yrs
7. What type of software your company develops? (In case of commercial use)

A. Custom software B. Product software C. Both D. Others ___________

1. Are the resources mapped with respect to project cost in distributed software development environment?
2. Very Unlikely B. Unlikely C. Neutral D. Likely E. Very Likely
3. Do the resources have sufficient technical trainings in terms of project management to run and execute the distributed softwares?
   1. Very Unlikely B. Unlikely C. Neutral D. Likely E. Very Likely
4. Do the resources have appropriate trainings working in a culturally diversified background?
   1. Very Unlikely B. Unlikely C. Neutral D. Likely E. Very Likely
5. Do the resources have appropriate trainings in working in geographically time, space project environment?
6. Very Unlikely B. Unlikely C. Neutral D. Likely E. Very Likely
7. Does your project support shift-handover environment in distributed development
8. Very Unlikely B. Unlikely C. Neutral D. Likely E. Very Likely
9. In distributed software development environment, do you use any project management techniques to bring project on track when there are time and cost over-runs?
10. Very Unlikely B. Unlikely C. Neutral D. Likely E. Very Likely
11. When project tasks are not completed, is there a plan in distributed environment to manage cost overruns?
12. Very Unlikely B. Unlikely C. Neutral D. Likely E. Very Likely
13. Is there a plan in distributed environment to re-allocate resources, considering the time and cost, when resources are not performing as planned?
14. Very Unlikely B. Unlikely C. Neutral D. Likely E. Very Likely
15. Is there someone assigned to monitor project variances and report weekly status updates in distributed teams?
16. Very Unlikely B. Unlikely C. Neutral D. Likely E. Very Likely
17. Does each task has a measurable plan which identifies clearly when the task is completed?
18. Very Unlikely B. Unlikely C. Neutral D. Likely E. Very Likely
19. Are costs and budget of the project monitored and forecasted by distribution teams?
20. Very Unlikely B. Unlikely C. Neutral D. Likely E. Very Likely
21. Have distributed teams a suitably detailed, up-to-date and shared schedule in place?
22. Very Unlikely B. Unlikely C. Neutral D. Likely E. Very Likely

1. Is the schedule being followed by the distributed project teams?
2. Very Unlikely B. Unlikely C. Neutral D. Likely E. Very Likely
3. Are the right resources available to the project to operate in different geographical locations and organizational boundaries?
4. Very Unlikely B. Unlikely C. Neutral D. Likely E. Very Likely
5. Are adequate resources provided for planning the software project?
6. Very Unlikely B. Unlikely C. Neutral D. Likely E. Very Likely
7. Are the resources being provided with the necessary training and tools to complete the project work according to their roles and responsibilities?
8. Very Unlikely B. Unlikely C. Neutral D. Likely E. Very Likely
9. Do the resources have a clear understanding of the project success criteria, goals/objectives, and current scope?
10. Very Unlikely B. Unlikely C. Neutral D. Likely E. Very Likely
11. Do the resources have a clear understanding of their assigned tasks, and due dates/milestones?
12. Very Unlikely B. Unlikely C. Neutral D. Likely E. Very Likely
13. Are the project’s actual results (e.g. Time and cost) compared with their original estimates in the software plans?
14. Very Unlikely B. Unlikely C. Neutral D. Likely E. Very Likely
15. Is corrective action taken when results differ significantly from the project’s software plans?
16. Very Unlikely B. Unlikely C. Neutral D. Likely E. Very Likely
17. Is there someone assigned for tracking the work including schedule and budget?
18. Very Unlikely B. Unlikely C. Neutral D. Likely E. Very Likely
19. Are regular risk reviews undertaken, as identified in the risk management plan, resulting in appropriate risk updates?
20. Very Unlikely B. Unlikely C. Neutral D. Likely E. Very Likely
21. Do you use automated tools to manage risks?

A. Yes B. No

1. If yes, name the tools you use to manage risks?

______________________________________________________________________________

1. If yes, how do you manage these risks?

______________________________________________________________________________

1. Which project management tools do you use in distributed environment? Please specify

____________________________________________________________________________
